# Relationship between preinduction electroencephalogram patterns and propofol sensitivity in adult patients

**DOI:** 10.1007/s10877-024-01149-y

**Published:** 2024-04-02

**Authors:** Seungpyo Nam, Seokha Yoo, Sun-Kyung Park, Youngwon Kim, Jin-Tae Kim

**Affiliations:** 1 Department of Anesthesiology and Pain Medicine, Seoul National University Hospital, Seoul National University College of Medicine, Seoul, Republic of Korea; 2 Department of Anesthesiology and Pain Medicine and Anesthesia and Pain Research Institute, Yonsei University College of Medicine, Seoul, Republic of Korea

**Keywords:** Band power, Induction dose of propofol, Preinduction electroencephalogram (EEG), Propofol requirement, Total intravenous anesthesia (TIVA)

## Abstract

**Purpose:**

To determine the precise induction dose, an objective assessment of individual propofol sensitivity is necessary. This study aimed to investigate whether preinduction electroencephalogram (EEG) data are useful in determining the optimal propofol dose for the induction of general anesthesia in healthy adult patients.

**Methods:**

Seventy healthy adult patients underwent total intravenous anesthesia (TIVA), and the effect-site target concentration of propofol was observed to measure each individual’s propofol requirements for loss of responsiveness. We analyzed preinduction EEG data to assess its relationship with propofol requirements and conducted multiple regression analyses considering various patient-related factors.

**Results:**

Patients with higher relative delta power (*ρ* = 0.47, *p* < 0.01) and higher absolute delta power (*ρ* = 0.34, *p* = 0.01) required a greater amount of propofol for anesthesia induction. In contrast, patients with higher relative beta power (*ρ* = -0.33, *p* < 0.01) required less propofol to achieve unresponsiveness. Multiple regression analysis revealed an independent association between relative delta power and propofol requirements.

**Conclusion:**

Preinduction EEG, particularly relative delta power, is associated with propofol requirements during the induction of general anesthesia. The utilization of preinduction EEG data may improve the precision of induction dose selection for individuals.

## Introduction

Propofol is one of the most commonly used intravenous anesthetics to induce general anesthesia in patients. It offers several advantages, including rapid onset, short duration of action, and robust safety profiles across various age groups [1]. One of the challenges in using propofol is determining the precise induction dose for individual patients to ensure adequate sedation while minimizing the risk of potential adverse events. Propofol often causes hypotension during induction by decreasing systemic vascular resistance and, to a lesser extent, myocardial depression [2–4]. As the incidence and severity of propofol-induced hypotension are dose-dependent [4–6], the risk of adverse outcomes associated with intraoperative hypotension, such as acute kidney injury, myocardial damage, morbidity, and even death, may increase if an excessive dose of propofol is administered [7–9]. Conversely, the use of an insufficient induction dose of propofol can lead to patient awareness, often resulting in severe mental health sequelae such as posttraumatic stress disorder (PTSD) [10, 11].

The current propofol dosing guidelines recommend administering a bolus of 1-2.5 mg/kg of propofol or initiating a continuous infusion with a plasma (Marsh model) or effect-site (Schnider model) propofol target concentration of 4–6 µg/ml when using a target controlled infusion (TCI) device to induce general anesthesia in healthy adult patients [12]. However, anesthesiologists often adjust the induction dose based on various factors, including age, sex, weight, health status, current medication, and coadministered drugs, recognizing the interpersonal variability in response to propofol [13–16]. Recent studies have reported discordance between the recommended induction dose range in the guidelines and the actual amounts of propofol administered to patients in clinical practice [4, 17, 18]. In particular, a multicenter study conducted by Schonberger et al. examined propofol dosing practices for geriatric patients (> 65 years) in the United States and revealed that approximately two-thirds of the studied patients received propofol induction doses exceeding the recommended dosage range, regardless of age and comorbidities [19]. Schonberger et al. further highlighted a potential concern in current propofol dosing practices, revealing a significant independent association between the propofol induction dose and the likelihood of preincision severe hypotension (MAP ≤ 55 mmHg) [20].

A recent study by Kweon et al. proposed the potential use of preinduction electroencephalogram (EEG) for predicting the optimal induction dose of propofol [21]. EEG monitoring, which measures the brain’s electrical activity, is essential in modern general anesthesia. It is widely used to assess sedation levels in patients, allowing clinicians to titrate the anesthetic dosage in real time as needed. Although many studies have confirmed the advantages of intraoperative EEG monitoring in perioperative management, the potential effectiveness of preinduction EEG monitoring has yet to be determined. Kweon et al. conducted a quantitative analysis of the full-scalp EEG of healthy patients undergoing dental procedures with propofol sedation prior to induction. They explored the relationship between the EEG patterns and propofol requirements using canonical correlation analysis. Their findings revealed that the power of delta oscillation in the frontal region provides valuable information regarding propofol requirements. Given that EEG reflects the functional status of the brain, where propofol exerts its sedation effect, it is reasonable to consider the possibility of a relationship between baseline brain activity and propofol sensitivity.

We conducted a prospective observational study to examine the relationship between preinduction EEG and propofol requirements for inducing unresponsiveness. In contrast to the previous study by Kweon et al., we used a simplified four-channel EEG monitoring device, the SedLine® Brain Function Monitor, instead of the conventional full-head scalp EEG monitoring to collect EEG data. Our goal was to validate the applicability of readily available EEG monitors in the operating room for predicting optimal propofol doses. Additionally, we performed multiple regression analysis to evaluate the independent associations between various patient characteristics and propofol requirements, aiming to identify the key factors contributing to the variability in optimal propofol induction doses.

## Method

### Study design

This prospective observational study received approval from the Institutional Review Board of Seoul National University Hospital (IRB number: 2008-082-1148) and was registered on ClinicalTrials.gov (registered IC: NCT04520503). The study involved healthy adults aged 20 to 89 years who were scheduled for elective noncardiac surgery under general anesthesia at Seoul National University Hospital from May 2019 to February 2022. The exclusion criteria included age under 20 years or over 90 years, American Society of Anesthesiologists (ASA) physical status IV or higher, body mass index (BMI) over 30 kg/m^2^, history of any neurological or psychiatric disorders, and significant cardiovascular, respiratory, renal, or hepatic disease. In addition, patients with factors that might interfere with the placement of the EEG sensors on the forehead were excluded.

### Data collection and the study protocol

Seventy patients were enrolled, and written informed consent was obtained before surgery. Baseline demographic data, including patient history, long-term medication use, and other comorbidities, were obtained from electronic medical records. Routine monitors, including noninvasive blood pressure, electrocardiograms, and pulse oximeters, were applied in the operating room. The patients were not premedicated before induction to obtain baseline EEG data in the operating room. Continuous EEG monitoring was conducted using the SedLine® Brain Function Monitor (Masimo Corporation, Irvine, California, USA), with EEG electrodes placed on each patient’s forehead according to the manufacturer’s instructions. Four active EEG leads were positioned at Fp1, Fp2, F7, and F8, and the ground electrode was positioned at Fz, with the reference electrode 1 cm posterior along the midline. Before electrode placement, the patients’ foreheads were cleaned using an alcohol swab to minimize impedance.

The patients were instructed to close their eyes and remain still for approximately two minutes to establish a baseline EEG. Initial processed EEG indices, including the patient status index (PSI), 95% spectral edge frequency (SEF), and suppression ratio (%), were recorded. Total intravenous anesthesia (TIVA) was administered using 2% propofol (Fresofol MCT inj 2%) and 20 µg/ml diluted remifentanil (Ultiva inj 1 mg) via a target-controlled infusion pump (Orchestra® Base Primea and Module DPS, Fresenius Kabi AG, Homburg, Germany). The patients were preoxygenated with 6 L/min oxygen via a facial mask, and a bolus of 0.5 mg/kg lidocaine (Huons Lidocaine HCl inj 1%, Huons Co., Seongnam, Korea) was administered before propofol infusion to alleviate injection pain. The modified Marsh model was chosen for propofol infusion because of its simplicity and sole reliance on total body weight as a covariate for dose and infusion rate calculations [22]. A slow induction method was used to monitor gradual changes in the participants’ responsiveness throughout the induction period. The initial effect-site target concentration (*Ce*) of propofol was set at 1 µg/ml and incrementally increased by 0.5 µg/ml until the patients became unresponsive. The patients were evaluated at each time point when the plasma concentration of propofol (*Cp*) and the estimated effect-site concentration of propofol (*Ce*), which were calculated and displayed in real time on the TCI device, reached the predetermined *Ce* value. Patients were prompted to open their eyes with a gentle tap on their shoulders. If the patients failed to respond, their eyelash reflexes were further checked to confirm their unresponsiveness. The effect-site concentration (*Ce*) of propofol at loss of responsiveness was defined as the propofol requirement for anesthesia induction. Subsequently, remifentanil infusion commenced with an effect-site target concentration of 2 ng/ml (Minto model), and a bolus of 0.6 mg/kg rocuronium was administered to prepare for endotracheal intubation. Following intubation, an anesthesiologist manually adjusted the propofol and remifentanil infusion doses to maintain stable hemodynamics and PSI within the range of 20–50.

### Electroencephalogram analysis

The SedLine® device collected and provided raw EEG data for retrospective analysis. The data can be exported to a European Data Format (EDF) file via a USB port. Raw EEG data obtained from the SedLine® device must be analyzed with care, as the initial EEG display setting of the device directly affects the EEG recording [23]. The display feed speed determines the sampling rate of the EEG, and the display amplitude resolution affects the quality and quantization of the recorded EEG. Therefore, our institution has standardized the display feed speed at 30 mm/s and amplitude at 25 µV/mm for all SedLine® devices, ensuring a sampling rate of 178 Hz and minimizing EEG distortions.

The exported raw EEG data were visualized and processed using the EEGLAB toolbox for MATLAB (MathWorks, Inc., Natick, Massachusetts, United States). The initial segments of continuous EEGs before propofol administration were isolated for analysis. These initial segments, approximately two minutes long, were defined as the preinduction EEG. For preprocessing, we removed the DC offsets and applied a bandpass filter (0.5–40 Hz) to eliminate noise signals. Subsequently, the preinduction EEGs were visually inspected, and a 10-second artifact-free time window was manually selected.

For the EEG spectral analysis, we utilized the multitaper spectral estimation method to compute the average band power at each channel. The average band power reflects the contribution of a specific frequency band to the overall power of the signal. The absolute and relative powers of the delta (0.5-4.0 Hz), theta (4.0–8.0 Hz), alpha (8.0–13.0 Hz), and beta (13.0–30.0 Hz) frequency bands were calculated from the preinduction EEGs at each channel using the MNE-Python package for Python [24]. Additionally, the average frequency of each electrode was computed using the Signal Processing Toolbox for MATLAB (MathWorks, Inc., Natick, Massachusetts, United States). These computed values were averaged across the four channels (Fp1, Fp2, F7, and F8) to obtain simplified representations of the neurophysiological characteristics of the frontal area.

### Statistical analysis

This study was designed as an exploratory pilot study; therefore, a formal sample size was not determined. Kweon et al. previously published a paper related to our topic of interest with a sample size of 30 [21]. We increased the sample size to 70 patients, including 10 patients in each decade, from their 20 to 80 s, to encompass a broader range of age groups.

All data are presented as the mean (SD) or number of patients (%). The patient demographic data included all 70 participants. However, one individual was excluded from further analysis as an extreme outlier to ensure the robustness and accurate interpretation of the results. The relationship between the propofol requirement and patient characteristics, such as demographic parameters, baseline vital signs, and EEG indices, was assessed using Spearman’s rank correlation coefficient (*ρ*), taking into account the skewed distribution of the propofol requirement. Simple linear regressions were used to explore the relationship between the relative powers of the specific frequency bands and the propofol requirement. Among the EEG parameters, relative delta power exhibited the strongest correlation with the propofol requirement. We constructed a multiple linear regression model (Model I) to evaluate the independent association between relative delta power and the propofol requirement. Other variables that displayed a relatively strong correlation with the propofol requirement (*ρ* > 0.4 or *ρ* < -0.4) were considered covariates and selected using a stepwise approach. Additionally, we constructed another multiple linear regression model (Model II) without EEG components. These two models were compared using an analysis of variance test and the Akaike information criterion (AIC) to assess the impact of adding EEG variables to the regression model. Normality was checked using the Kolmogorov‒Smirnov and Shapiro‒Wilk tests, and a collinearity diagnostic was conducted to evaluate the interrelation of selected predictors in the models.

To ascertain whether an adequate amount of propofol was administered to each patient during anesthesia induction, changes in hemodynamics and processed EEG indices, such as the PSI, SEF, and SR, were analyzed at three different time points during induction (T1: loss of responsiveness, T2: immediately after intubation, T3: two minutes after intubation). Patients were divided into four groups based on their propofol requirements: Group A (*Ce* of propofol ≤ 2.5 µg/ml, *n* = 15), Group B (*Ce* of propofol = 3.0 µg/ml, *n* = 28), Group C (*Ce* of propofol = 3.5 µg/ml, *n* = 12), and Group D (*Ce* of propofol ≥ 4.0 µg/ml, *n* = 14). One-way analysis of variance and the Kruskal‒Wallis test were used to analyze the differences in HR and MBP percentages from baseline, as well as EEG indices, including the PSI, SEF, and SR, among the groups, as appropriate.

Microsoft Excel was used for data summarization and organization. All the statistical analyses were performed with RStudio (Posit Software, PBC, Boston, MA; version 2022.12.0.353). A *p* < 0.05 was considered to indicate statistical significance.

## Results

**Fig. 1:**
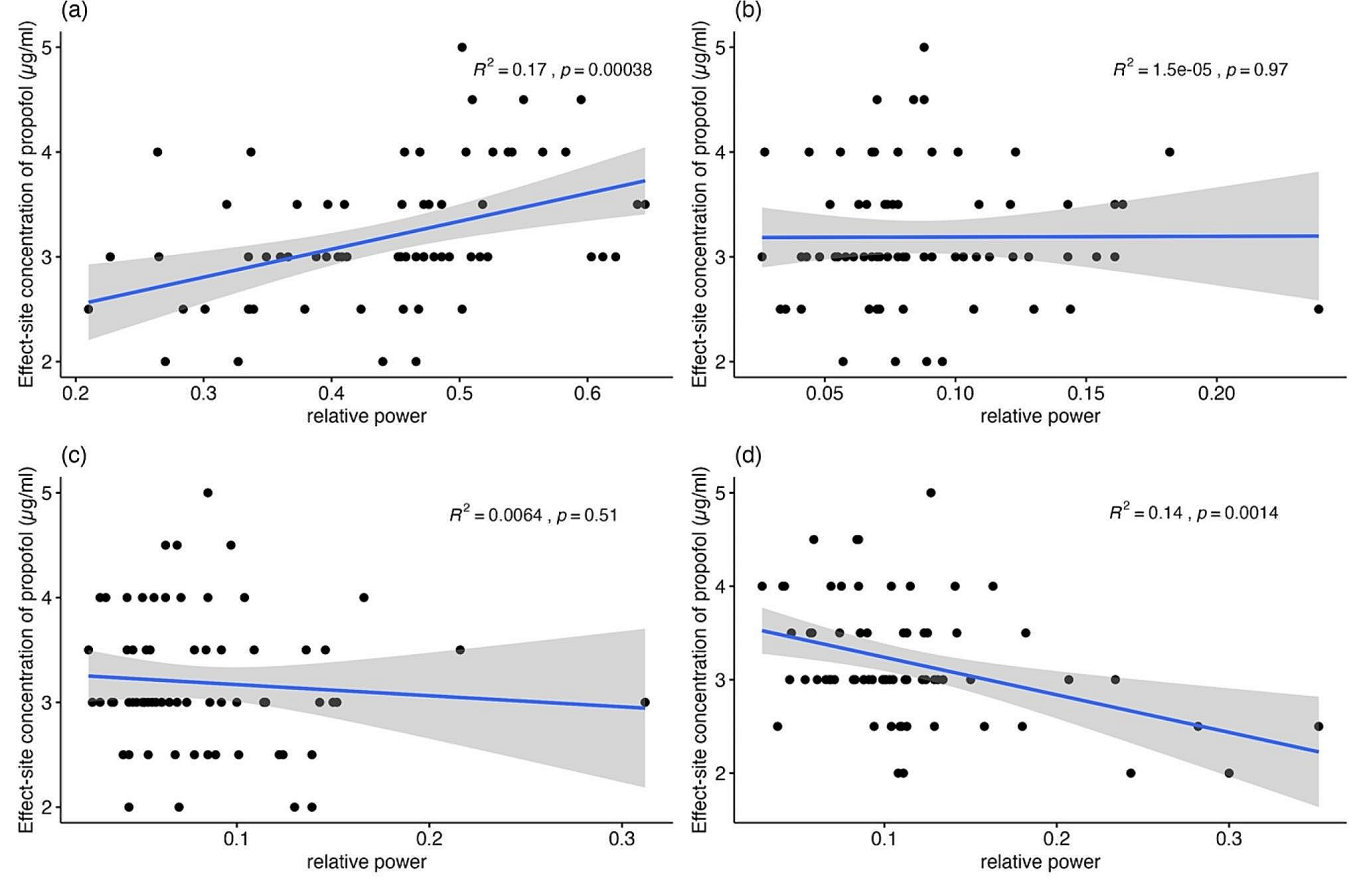
Regression (95% CI) analysis showing the relationship between the propofol requirement and relative powers of specific bandwidths: (**a**) relative delta power; (**b**) relative theta power; (**c**) relative alpha power; (**d**) relative beta power

The baseline patient characteristics are presented in Table 1. The relationship between propofol requirement and patient characteristics were analyzed by calculating correlation coefficients, as summarized in Table 2. Significant positive correlations were found between the propofol requirement and the relative delta power (*ρ* = 0.47, *p* < 0.01) and absolute delta power (*ρ* = 0.34, *p* = 0.01). Conversely, negative correlations were observed with relative beta power (*ρ* = -0.33, *p* < 0.01), age (*ρ* = -0.48, *p* < 0.01), ASA physical status (*ρ* = -0.41, *p* < 0.01), and BMI (*ρ* = -0.46, *p* < 0.01). Other parameters did not show a significant correlation with the propofol requirement or exhibited relatively weak correlations. Simple linear regression was used to determine whether the relative powers of specific frequency bands significantly predicted the propofol requirement (Fig. 1). The regression results revealed that relative delta power and relative beta power accounted for 17% and 14% of the variation in the propofol requirement, respectively [F(1,67) = 14.01, *p* < 0.01; F(1,67) = 11.18, *p* < 0.01].

**Table 1:** Patient characteristics, means (SD) or counts (%)

Number of patients	*n* = 70
Age (yr)	54.1 (19.6)
Sex, male/female	35 (50)/35 (50)
Height (cm)	163.8 (8.9)
Weight (kg)	63.7 (11.4)
BMI (kg/m^2^)	23.7 (2.9)
ASA physical status, I/II/III	19 (27.1)/48 (68.6)/3 (4.3)
Baseline vital signs	
Heart rate (/min)	70.2 (12.9)
SBP (mmHg)	152.5 (27.7)
DBP (mmHg)	77.9 (13.5)
MBP (mmHg)	102.2 (16.2)
SpO2 (%)	98.6 (1.6)

ASA: American Society of Anesthesiologists physical status classification.

**Table 2:** Correlation coefficient between patient characteristics and the propofol requirement

	Propofol Requirement		
	*ρ*		*p* value
Age, yr	-0.48		< 0.01
ASA physical status	-0.41		< 0.01
BMI, kg/m^2^	-0.46		< 0.01
Initial vital sign			
HR	0.10		0.40
SBP	-0.19		0.12
DBP	-0.06		0.63
MBP	-0.13		0.30
SpO2	0.29		0.01
Initial processed EEG indices			
PSI	-0.08		0.54
SEFL	-0.18		0.15
SEFR	-0.05		0.72
Baseline EEG indices			
Relative delta power	0.47		< 0.01
Relative theta power	0.05		0.68
Relative alpha power	-0.08		0.49
Relative beta power	-0.33		< 0.01
Absolute delta power	0.34		0.01
Absolute theta power	0.25		0.04
Absolute alpha power	0.12		0.31
Absolute beta power	-0.10		0.42
mean frequency	-0.21		0.08

PSI: Patient status index

SEFL: spectral edge frequency of the left frontal area

SEFR: spectral edge frequency of the right frontal area.

Stepwise multiple regression analysis was conducted to assess whether the propofol requirement could be estimated using preoperative variables, including relative delta power, age, BMI, and ASA physical status. The results indicated that relative delta power, age, and BMI were significant predictors of the propofol requirement, with relative delta power having a positive effect, while age and BMI had a negative effect (Table 3). This regression model (Model I) explained 47% of the variance in the propofol requirement. To emphasize the importance of the preinduction EEG parameter in predicting the propofol requirement, another stepwise multiple regression model (Model II) was constructed by excluding the relative delta power from selected covariates. Although the explanatory power of Model II was similar to that of Model I, Model I was considered to be a better fit due to its lower Akaike information criterion (AIC) value compared to Model II.

**Table 3:** Comparison between regression models

	β			F (df)	*p* value	R^2^	Adj R^2^	AIC
	Relative delta power	Age	BMI					
SLR	2.67	-	-	14.01 (1,67)	< 0.01	0.17	0.16	126.61
Model I	1.49	-0.01	-0.08	19.38 (3,65)	< 0.01	0.47	0.45	99.62
Model II	-	-0.01	-0.09	24.26 (2,66)	< 0.01	0.42	0.41	103.68

SLR: Simple linear regression model

Model I: relative delta power, age, and BMI as covariates

Model II: age and BMI as covariates

AIC: Akaike information criterion

Processed EEG indices, including the PSI, SEF, and SR, were monitored to assess the depth of sedation throughout the induction period. The average PSI at the time of loss of responsiveness was 62.4 ± 21.1, indicating that it was outside the optimal hypnotic state for anesthesia. However, the average PSI decreased to 40.3 ± 10.2 at the time of intubation, thereby maintaining the optimal hypnotic state.

To evaluate whether the propofol effect-site concentration (*Ce*) required to achieve a loss of responsiveness for each patient was appropriate for stable induction, the studied patients were categorized into four groups based on their propofol requirements, and changes in vital signs and EEG indices were compared. Table 4 summarizes the mean processed EEG indices for each group and the percent changes in heart rate and mean blood pressure. The results showed no significant difference in the percent change in heart rate or mean blood pressure among the groups during induction. Additionally, the processed EEG indices were not significantly different among the groups at the time of intubation, but these values were significantly different two minutes after intubation.

**Table 4:** The mean (SD) processed EEG indices and vital sign changes during induction

		Total (*n* = 69)		Group A (*n* = 15)		Group B (*n* = 28)		Group C (*n* = 12)		Group D (*n* = 14)		*p* value	
T1	*Target Ce*												
	Propofol (µg/ml)		3.2 (0.6)		2.4 (0.2)		3.0 (0)		3.5 (0)		4.2 (0.3)		< 0.01
	Remifentanil (ng/ml)		-		-		-		-		-		-
	*EEG indices*												
	PSI		62.4 (21.1)		68.1 (19.3)		62.5 (20.3)		59.7 (23.0)		58.8 (24.1)		0.56
	SEFL (Hz)		14.3 (6.4)		19.4 (6.4)		13.3 (5.8)		13.6 (6.7)		11.5 (4.6)		< 0.01
	SEFR (Hz)		14.2 (7.0)		19.9 (6.4)		13.0 (5.6)		12.6 (7.4)		12.2 (7.4)		< 0.01
	SR (%)		0.1 (0.7)		0.0 (0.0)		0.2 (0.8)		0.3 (1.2)		0.0 (0.0)		0.57
	*Changes in vital sign*												
	△HR (%)		-5.0 (14.8)		-6.7 (9.2)		-5.7 (17.8)		-2.6 (18.3)		-4.0 (10.3)		0.51
	△MBP (%)		-13.4 (11.6)		-11.1 (14.3)		-13.4 (12.2)		-15.8 (9.7)		-13.5 (9.0)		0.78
T2	*Target Ce*												
	Propofol (µg/ml)		3.2 (0.6)		2.4 (0.2)		3.0 (0)		3.5 (0.1)		4.0 (0.5)		< 0.01
	Remifentanil (ng/ml)		2.1 (0.5)		2.0 (0)		2.0 (0)		2.3 (1.2)		2.0 (0)		0.19
	*EEG indices*												
	PSI		40.3 (10.2)		42.5 (9.7)		40.7 (7.0)		38.3 (9.8)		38.9 (15.8)		0.20
	SEFL (Hz)		11.8 (4.6)		13.0 (4.9)		11.4 (5.0)		11.5 (4.2)		11.4 (4.1)		0.51
	SEFR (Hz)		12.5 (4.1)		13.6 (4.2)		12.0 (4.6)		12.0 (4.4)		12.9 (2.7)		0.66
	SR (%)		0.4 (1.5)		0.7 (2.7)		0.4 (1.1)		0.3 (1.2)		0.0 (0.0)		0.67
	*Changes in vital sign*												
	△HR (%)		20.6 (25.2)		26.9 (32.7)		19.2 (23.9)		17.7 (22.9)		19.2 (22.0)		0.92
	△MBP (%)		6.3 (22.6)		1.8 (23.3)		10.2 (21.4)		3.8 (24.1)		5.4 (24.4)		0.61
T3	*Target Ce*												
	Propofol (µg/ml)		3.0 (0.6)		2.3 (0.3)		2.9 (0.3)		3.3 (0.3)		3.5 (0.7)		< 0.01
	Remifentanil (ng/ml)		2.4 (0.7)		2.1 (0.4)		2.4 (0.7)		2.5 (0.8)		2.5 (0.7)		0.37
	*EEG indices*												
	PSI		40.4 (8.2)		44.6 (6.9)		42.9 (7.9)		35.4 (6.7)		35.7 (7.1)		< 0.01
	SEFL (Hz)		14.8 (3.8)		15.0 (3.3)		16.8 (3.2)		12.5 (4.3)		12.8 (2.8)		< 0.01
	SEFR (Hz)		14.6 (3.6)		14.9 (2.8)		16.3 (3.8)		13.1 (3.3)		12.4 (2.4)		< 0.01
	SR (%)		0.0 (0.0)		0.0 (0.0)		0.0 (0.0)		0.0 (0.0)		0.0 (0.0)		-
	*Changes in vital sign*												
	△HR (%)		6.9 (18.8)		13.0 (23.3)		4.7 (17.7)		2.1 (16.1)		8.8 (17.8)		0.42
	△MBP (%)		-8.3 (17.2)		-7.5 (18.9)		-8.1 (15.7)		-15.2 (17.5)		-3.4 (18.0)		0.39

Group A: Ce of propofol ≤ 2.5 µg/ml

Group B: Ce of propofol = 3.0 µg/ml

Group C: Ce of propofol = 3.5 µg/ml

Group D: Ce of propofol ≥ 4.0 µg/ml

T1: loss of responsiveness

T2: Immediately after intubation

T3: 2 min after intubation

PSI: patient status index; SEFL: spectral edge frequency, left frontal area

SEFR: spectral edge frequency, right frontal area; SR: suppression ratio (%)

## Discussion

In this study, we investigated whether preinduction EEG of the frontal area, as measured by the SedLine® brain function monitor, could be useful for determining the optimal propofol dose for the induction of general anesthesia in healthy adults. We observed the *Ce* of propofol at the point of loss of responsiveness, defined as the propofol requirement, and examined its relationship with various patient factors, including preinduction EEG features. Among the EEG features, the relative delta and beta powers in the frontal region demonstrated the strongest correlation with the propofol requirement. These findings suggest that preinduction EEG has the potential to serve as a biomarker for estimating an individual’s sensitivity to propofol in the brain, providing valuable insights for determining a more precise induction dosage.

The variability in an individual’s response to propofol poses a challenge in determining the optimal induction dose. Our study participants lost responsiveness at an average *Ce* of 3.2 ± 0.65 µg/ml, with a wide range of *Ce* between 2.0 and 5.0 µg/ml. The current guidelines do not provide precise induction dosage, but rather offer a range of doses that anesthesiologists can adjust according to various factors, including patient characteristics, coadministered drugs, and clinical situations. In this study, age, ASA physical status, and BMI exhibited the most significant correlations with the propofol requirement, aligning with their common consideration in current clinical practice. Generally, elderly and frail patients tend to demonstrate increased sensitivity to propofol compared to young and healthy individuals due to physiological changes [13, 15, 25]. Patients with obesity also undergo physiological alterations, including an increase in cardiac output and a reduction in blood flow to adipose tissue, which enhances propofol sensitivity by increasing propofol delivery to the brain [22, 26–28]. Nonetheless, it is essential to acknowledge that relying solely on patient characteristics to estimate propofol requirements may overlook significant interindividual differences among individuals with similar attributes.

The evaluation of the brain may be crucial, but it is often neglected in dosage determination. Propofol exerts its sedative effect by positively modulating the inhibitory function of the neurotransmitter gamma-aminobutyric acid (GABA) through GABAA receptors in the brain [29]. Therefore, the functional status of the brain is likely to affect the pharmacodynamics of propofol. A study conducted by Laalou et al. demonstrated that propofol requirements vary depending solely on differences in cognitive function, even after controlling for other patient characteristics such as age [25]. Their results showed that a decrease in the preoperative cognitive status of patients reduced propofol requirements to maintain hypnosis, indicating that the functional status of the brain is an independent factor associated with propofol sensitivity. A recent study by Kweon et al. investigated the relationship between EEG patterns and propofol requirements [21]. They demonstrated that the delta power of the frontal area in preinduction EEG is strongly associated with the propofol requirements for induction. Consistent with their results, our study revealed that relative delta power exhibited the strongest correlation with the propofol requirement among the various EEG parameters, followed by relative beta power. Kweon et al. did not provide detailed information regarding the relationship between delta power and the propofol requirement, nor did they explicitly discuss the practical implications of their findings in current propofol dosing practices. However, based on these results, we can hypothesize that patients with a lower relative delta or a higher relative beta power in the frontal area become more sensitive to propofol, requiring less propofol to achieve unresponsiveness.

Although cognitive function and EEG patterns are distinct aspects of brain functional status, they are both associated with brain age. Brain age is an estimate of the biological age of the brain based on its structural and functional characteristics. Brain age is closely related to chronological age, but they are not the same. Therefore, the discrepancy between brain age and chronological age may contribute to varying degrees of propofol sensitivity among individuals of similar ages [30, 31]. Brain age is typically estimated by examining structural changes using magnetic resonance imaging (MRI), but it can also be estimated using EEG. The distribution of band power in the resting state tends to change with age, shifting from slow to fast oscillatory rhythms [32–37]. Consequently, patients with older brains tend to display a lower distribution of delta oscillations but a higher distribution of beta oscillations. Our results confirmed that integrating relative delta power into predictions significantly improved the predictive accuracy compared to predictions based solely on age and BMI. This finding implies that EEG patterns, particularly relative delta power, may play a crucial role in predicting propofol sensitivity.

The positive correlation between relative delta power and the propofol requirement may seem paradoxical in healthy adults, given the close association of delta waves with sleep and general anesthesia [38, 39]. However, delta waves are also present in an awake state, particularly during early developmental stages, and their prominence tends to decrease with age due to changes in the brain’s anatomy and neurophysiology [32]. Distinct oscillatory rhythms in different areas of the brain play specific roles. Previous studies have shown that delta activity increases in the frontal region during cognitive tasks that demand attention and concentration. An increase in delta oscillation appears to inhibit interference that could negatively affect task performance by deactivating interneurons and thalamocortical inputs [40–42]. Similarly, during anesthesia, an increase in delta oscillation may help maintain unconsciousness by phase-locking neuronal spiking, limiting cortical activity, and impeding long-range cortical communication [43]. These findings suggest that a higher delta power might enhance the ability to resist external stimuli, such as the sedative effect of propofol, and contribute to maintaining consciousness.

There are several limitations to our study. First, the study was conducted at a single center with a relatively small sample size. Future studies related to this topic should formally calculate sample sizes to determine the generalizability of the results to larger populations with increased confidence. Second, the study participants were exclusively healthy patients (ASA physical status ≤ 3) without known neurological or psychological conditions. This may limit the applicability of our findings to a more diverse patient population. Subsequent research should explore how various conditions, such as neurological disorders [44–48], emotional states [49, 50], and sleep deprivation [51, 52], influence the relationship between EEG patterns and propofol sensitivity. Third, this study used a simplified method for preprocessing the EEG data because of the limited number of electrodes, hindering the use of more advanced techniques for cleaning and enhancing raw EEG data before analysis. Finally, we exclusively conducted PSD analysis on the EEG data. A more comprehensive understanding of EEG data could be achieved by incorporating other quantitative analyses, such as examining brain connectivity and the phase lag index. Diverse patient populations and various quantitative analysis methods should be considered in future investigations, as well as the determination of an appropriate sample size to fully elucidate the relationship between preinduction EEG patterns and propofol requirements.

In conclusion, our study investigated the potential use of preinduction EEG as a predictive tool for determining propofol requirements during general anesthesia induction in healthy adults. Notably, significant associations were found between the propofol requirement and the relative delta and beta powers of the frontal area.
